# The Role of Glutathione in Age-Related Macular Degeneration (AMD)

**DOI:** 10.3390/ijms25084158

**Published:** 2024-04-09

**Authors:** Sylwia Brodzka, Jędrzej Baszyński, Katarzyna Rektor, Karolina Hołderna-Bona, Emilia Stanek, Natalia Kurhaluk, Halina Tkaczenko, Grażyna Malukiewicz, Alina Woźniak, Piotr Kamiński

**Affiliations:** 1 Division of Ecology and Environmental Protection, Department of Medical Biology and Biochemistry, Faculty of Medicine, Collegium Medicum in Bydgoszcz, Nicolaus Copernicus University in Toruń, M. Skłodowska-Curie St. 9, PL 85-094 Bydgoszcz, Poland; sylwia.brodzka@cm.umk.pl (S.B.); jedrzej.baszynski@cm.umk.pl (J.B.); karolina.holderna@cm.umk.pl (K.H.-B.); emilia.stanek@cm.umk.pl (E.S.); 2 Department of Biotechnology, Institute of Biological Sciences, Faculty of Biological Sciences, University of Zielona Góra, Prof. Z. Szafran St. 1, PL 65-516 Zielona Góra, Poland; k.rektor@wnb.uz.zgora.pl; 3 Institute of Biology, Pomeranian University in Słupsk, Arciszewski St. 22 B, PL 76-200 Słupsk, Poland; natalia.kurhaluk@upsl.edu.pl (N.K.); halina.tkaczenko@upsl.edu.pl (H.T.); 4 Department of Eye Diseases, University Hospital No. 1, Faculty of Medicine, Collegium Medicum in Bydgoszcz, Nicolaus Copernicus University in Toruń, M. Skłodowska-Curie St. 9, PL 85-092 Bydgoszcz, Poland; g.malukiewicz@cm.umk.pl; 5 Department of Medical Biology and Biochemistry, Faculty of Medicine, Collegium Medicum in Bydgoszcz, Nicolaus Copernicus University, M. Karłowicz St. 24, PL 85-092 Bydgoszcz, Poland; al1103@cm.umk.pl

**Keywords:** age-related macular degeneration, AMD, oxidative stress, glutathione (GSH), glutathione peroxidase (GPx), glutathione reductase (GR), glutathione S-transferase (GST), *GSTT1*, *GSTM1*

## Abstract

Age-related macular degeneration (AMD) is a chronic disease that usually develops in older people. Pathogenetic changes in this disease include anatomical and functional complexes. Harmful factors damage the retina and macula. These changes may lead to partial or total loss of vision. The disease can occur in two clinical forms: dry (the progression is slow and gentle) and exudative (wet—progression is acute and severe), which usually starts in the dry form; however, the coexistence of both forms is possible. The etiology of AMD is not fully understood, and the precise mechanisms of the development of this illness are still unknown. Extensive genetic studies have shown that AMD is a multi-factorial disease and that genetic determinants, along with external and internal environmental and metabolic-functional factors, are important risk factors. This article reviews the role of glutathione (GSH) enzymes engaged in maintaining the reduced form and polymorphism in *glutathione S-transferase theta-1* (*GSTT1*) and *glutathione S-transferase mu-1* (*GSTM1*) in the development of AMD. We only chose papers that confirmed the influence of the parameters on the development of AMD. Because GSH is the most important antioxidant in the eye, it is important to know the influence of the enzymes and genetic background to ensure an optimal level of glutathione concentration. Numerous studies have been conducted on how the glutathione system works till today. This paper presents the current state of knowledge about the changes in GSH, GST, GR, and GPx in AMD. GST studies clearly show increased activity in ill people, but for GPx, the results relating to activity are not so clear. Depending on the research, the results also suggest higher and lower GPx activity in patients with AMD. The analysis of polymorphisms in *GST* genes confirmed that mutations lead to weaker antioxidant barriers and may contribute to the development of AMD; unfortunately, a meta-analysis and some research did not confirm that connection. Unspecific results of many of the parameters that make up the glutathione system show many unknowns. It is so important to conduct further research to understand the exact mechanism of defense functions of glutathione against oxidative stress in the human eye.

## 1. Introduction 

Age-related macular degeneration (AMD) is a chronic eye disease that usually appears in people after the age of 50; however, in highly developed countries, it also occurs in younger people. AMD is an important cause of blindness in the USA and accounts for 54% of blindness in Caucasian patients, 4.4% in black patients, and 14.3% in Spanish patients. Today, among white people aged 40 and older, AMD is the most common cause of visual impairment and blindness in the USA [1]. According to the increasing life expectancy, it is expected that the frequency of AMD will increase [2,3,4,5]. The precise development mechanism of this illness is still unknown. Previous studies have shown that, due to pathological changes in the retinal pigment epithelium (RPE), the macula is damaged, which may lead to partial or complete loss of vision [6].

**Figure 1:**
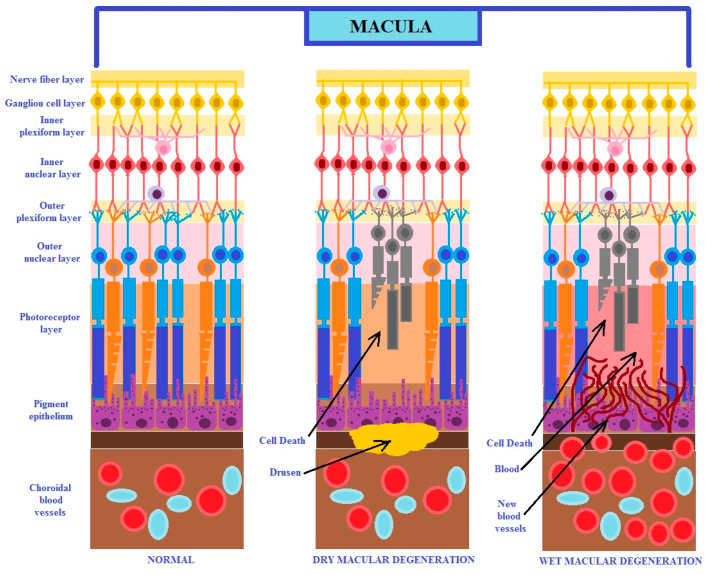
The figure shows the two different clinical forms of macular degeneration (modified from the work of Nayyar et al. (2020) [7]).

It is known that age-related macular degeneration occurs in two different pathophysiological forms: dry and exudative (known as wet or neovascular). The dry form of the disease is characterized by mild and gradual degeneration, and in the neovascular form, the disease has an acute and violent nature. Usually, the dry form transforms into the wet form (Figure 1) [7]. This disease can develop in only one eye, but with progression, it may affect both ones. It has been reported that both forms of AMD may occur simultaneously [2,3,4,5].

**Figure 2:**
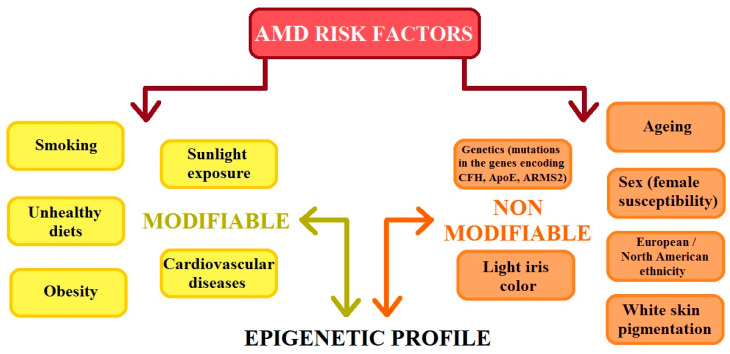
The figure shows the risk factors for age-related macular degeneration (AMD). CFH: complement H activity; ApoE: apolipoprotein E; ARMS2: age-related maculopathy susceptibility 2 (modified from the work of Hyttinen et al. (2023) [9]).

AMD is a heterogenic and multi-factorial disease that can also be influenced by numerous environmental and genetic risk factors [3,4,7], which significantly contribute to the development of the disease. Studies have confirmed that environmental factors such as smoking, a high cholesterol diet, carotenoids, vitamins A and E levels, zinc levels, age, sex, and the presence of heavy metal ions and chemicals slightly increase the risk of AMD. Previous studies have shown that there is no clear evidence that oxidative stress is a major factor in AMD development; however, reactive oxygen species (ROS) continue to be an important factor in the course of this disease [2,3,4,8]. AMD risk factors can be classified as modifiable and non-modifiable. The division of these groups of factors is illustrated in Figure 2. It is important that modifiable and non-modifiable AMD risk factors are mutually dependent on the epigenetic regulation of gene expression, which is mainly determined by the cellular epigenetic profile [9]. 

Four processes take part in the development of AMD: lipofuscinogenesis, drusogenesis, inflammation, and neovascularization [10]. As we described earlier, retinal cells are exposed to oxidative stress to a large extent due to the effects of light [2,3,4]. As a result of aging processes, the cells do not respond to mitogenic factors and lose their ability to proliferate. Studies have confirmed the presence of AGE-exposed genes (advanced glycation products) in RPE cells [11]. They are deposited in lysosomes in the form of lipofuscin (lipofuscinogenesis), which, in turn, is deposited underneath the RPE as a drusen (drusogenesis) [10]. As a result, this disturbs the metabolic exchange between the choroid and retina [12], weakening the activity of photoreceptors [13]. Then, low-intensity inflammatory processes begin, which increase the levels of acute-phase proteins, reducing total antioxidant status (TAS) [4,14]. The next stage is neovascularization, which occurs due to the secretion of proangiogenic factors during the para-inflammatory process. New capillaries come from the choroid and are formed under the retina in the macula area, where they should not occur [4]. As a result of all of these stages, photoreceptors are destroyed in the macula [2,3,4].

## 2. The Role of Reactive Oxygen Species (ROS)

The retina is exposed to light and consumes large amounts of oxygen when transforming light into vision. As a result, ROS production takes place [15]. ROS, as a group of compounds (hydrogen peroxide (H_2_O_2_), superoxide radical anions (O_2_*^−^), and hydroxyl radicals (*OH), react with other molecules and promote lipid peroxidation (LPO) [2,3,4]. These processes, involving ROS and LPO, are shown in Table 1. 

These various particle molecules can form in many ways, e.g., as a product of the respiratory chain in mitochondria, photochemical or enzymatic reactions, as an effect of exposure to UV light, exposure to ionizing radiation, or the influence of metal ions [2,3,4,16]. ROS play an important role in the regulation of many physiological processes by participating in intracellular signaling [16,20], but they can also cause serious damage to biomolecules via lipid peroxidation (autoxidation). ROS also attack structural and enzymatic proteins via the oxidation of amino acid residues, the formation of transverse bonds and protein aggregates, and proteolysis. The deactivation of key proteins can have serious consequences in important metabolic pathways. ROS can also react with nucleic acids. The inability of the cell to repair the damage incurred may lead to the death of the cell; alternatively, there may be mutations in the DNA, leading to the development of diseases [2,3,4,16].

It is known that the retina is the element that is most exposed to oxidative stress. This is due to its continuous exposure to radiation, high concentrations of O_2_, high levels of polyunsaturated fatty acids in the external photoreceptors, and the presence of many chromophores (lipofuscin, melanin, rhodopsin, and cytochrome C oxidase in the retina and retinal pigment epithelial cells (RPE) that also generate ROS in phagocytosis photoreceptor disks by RPE [4,21]. Molecular damage caused by ROS results in the formation and activity of advanced glycation products (AGEs). These products may affect cell DNA and increase the expression of genes that promote RPE cell aging [22]. These harmful factors are associated with the progression of AMD [2,4,23,24]. As a result of recognition system disorders, DNA repair genes are not able to efficiently repair DNA damage. In effect, changes accelerate the aging of the organism, leading to the dysfunction of cells and tissues. ROS formation is the result of normal, daily cellular metabolism, which is why aging is unavoidable. To protect molecules from the negative influence of ROS, cells have developed complex defense mechanisms [3,4,16]. According to these studies, photoreceptors and RPE cells are some of the most susceptible to oxidative stress damage. These non-proliferating postmitotic cells do not have any DNA damage detection systems at the cell cycle checkpoints [3,4]. 

The retinal pigment epithelium (RPE) performs a number of crucial functions, such as in the formation of an external retinal barrier, transport, retinoid retention, phagocytosis, the degradation of segmental photoreceptors, and protection against light and oxidative stress [25]. In this part of the eye, the dominant photoreceptors are cones. They are characterized by higher demand and energy production than rods, leading to higher oxygen requirements. In addition, rod cells and cones differ in their susceptibility to oxidative stress. Unfortunately, cones show greater sensitivity to free radicals [26]. 

The macula is also permanently exposed to high metabolic activity and oxidative stress due to the high partial pressure of choriocapillaris and polyunsaturated fatty acids (PUFAs), which is related to the external segments of the retina [2,3,4,27]. This is considered important in inducing drusen formation between RPE cells and Bruch’s membrane. Lipofuscin is a chromophore that serves as a primary RPE photo-oxide [28], which, after absorbing high energy photons (especially blue light), undergoes a series of photochemical reactions. These photochemical reactions lead to ROS formation, which, in turn, induces photochemical damage in the retina and RPE cells [2,3,4,29]. The role of auto-phagocytosis and the RPE homeostasis pathway plays an important role in the aging of cells and the effect of oxidative stress on AMD. Studies on the cellular and molecular pathways of autophagy showed that acute exposure to oxidative stress increased autophagy activity, while chronic exposure to oxidative stress reduced autophagy. Based on animal and human models, elevated levels of autophagy in the early AMD stage have been observed, with activity decreasing in late AMD. Moreover, it has been observed that the reduced effect of auto-phagocytosis makes RPE cells more susceptible to oxidative stress and that the autophagy system is enhanced to protect RPE cells from oxidative damage [25].

## 3. Antioxidant Defense 

To counteract the effects of oxidative stress, a response is triggered in two stages. The first stage is a reaction where the cytochrome P450 monooxygenase system and aldo-keto-reductase (AKR), carboxylesterases (CES), and epoxide hydrolase are involved. This action involves the oxidation and reduction of dangerous compounds. In the next phase, these products are coupled with hydrophilic molecules. The antioxidants involved in the second phase are divided into so-called “direct” and “indirect” categories. The “direct” group contains superoxide dismutase (SOD), glutathione (GSH), and thioredoxin reductase (Trx). The first two enzymes oxidize dangerous compounds and quickly regenerate themselves. The so-called “indirect” enzymes take part in the biosynthesis and regeneration of GSH and Trx. They also participate in the removal of oxidized compounds [11]. 

An antioxidant barrier cannot function without nuclear factor erythroid-2 related factor 2 (Nrf2). The antioxidants involved in the second phase are regulated by transcriptional master Nrf2/Keap1 (nuclear factor erythroid-2 related factor 2/Kelch Like ECH Associated Protein 1). This factor plays a major role in antioxidant response. It activates the transcription of the leucine zipper by binding to the antioxidant response element (ARE) within the promoter of the target genes. Its role is to maintain redox homeostasis in the cell [2,30,31]. Nrf2 regulates the action of both “direct” and “indirect” enzymes [32,33]. The “direct” group is created by SOD, glutathione peroxidase (GPX), catalase (CAT), and peroxiredoxins (PRDXs). This group of “indirect” enzymes contains transketolase (TKT), glucose-6-phosphate dehydrogenase (G6PD), glyceraldehyde-3-phosphate dehydrogenase (GAPDH), glutathione-redox cycle enzymes (glutathione reductase (GSR), glutathione synthetase (GSS)), selenoproteins, and thioredoxin-related enzymes (thioredoxin (TXN), sulfiredoxin (SRXN)) [34]. 

## 4. Glutathione Function in AMD 

Glutathione is the most important low-molecular antioxidant from the thiol group [35,36,37]. It exists in two forms: oxidized glutathione disulfide (GSSG), which is reduced as a tripeptide gamma-glutamyl-cysteinyl-glycine (GSH). The GSH thiol group of cysteine can donate a reducing equivalent (H^+^ + e^−^) to different unstable molecules (e.g., ROS). After donation, glutathione becomes reactive but can easily react with other reactive glutathione and, in effect, may create the glutathione disulfide (GSSG) form [38]. These two molecules (GSH and GSSG) form a redox buffer system [39]. Analyzing the status of this system allows us to determine the status of the oxidative state in the intracellular environment. The GSH:GSSG ratio is specific to intracellular compartments [39,40]. The concentration of GSH in cells depends on their type and ranges from 5 to 10 mM [36,37], while the level of GSH in the serum is much lower (20 µM) because the oxidized form (GSSG) predominates (2GSH + R_2_O_2_ → GSSG + 2ROH) [37]. 

Glutathione occurs in eukaryotic cells (both animals and plants) [35,36,37]. In the case of physiological pH, GSH has functional groups (one positively charged amino group and two negatively charged carboxyl groups) that allow for reactions with various macromolecules [41,42]. Along with ascorbate, albumin, cysteine, uric acid, creatinine, bilirubin, melanin, beta-carotene, and others, glutathione is one of the most important antioxidants in the human body. To protect cells from oxidative stress, glutathione competes with other antioxidants when reducing ROS. GSH is also involved in different vital functions. This molecule plays a role in xenobiotic metabolism by forming S-Nitroso-glutathione (GSNO), which can be a reservoir for nitric-oxide (NO) [42] and plays a role in the regulation of blood pressure. GSH is necessary for the transformation of prostaglandin H2 into prostaglandin D2, which is also important in leukotriene synthesis. It has been confirmed that GSH inhibits infection caused by influenza virus [43]. On the other hand, a study during the SARS-CoV-2 pandemic showed that cellular glutathione metabolism and redox function are impaired in this disease [44]. 

It is essential to keep GSH in cells at an appropriate level because when it is significantly lowered, the accumulation of H_2_O_2_ may occur, which results in cell damage [35,41]. In effect, low GSH concentrations may lead to the development of various diseases, such as neurodegeneration, mitochondrial dysfunction, and even cancer [38,42,45,46]. 

**Figure 3:**
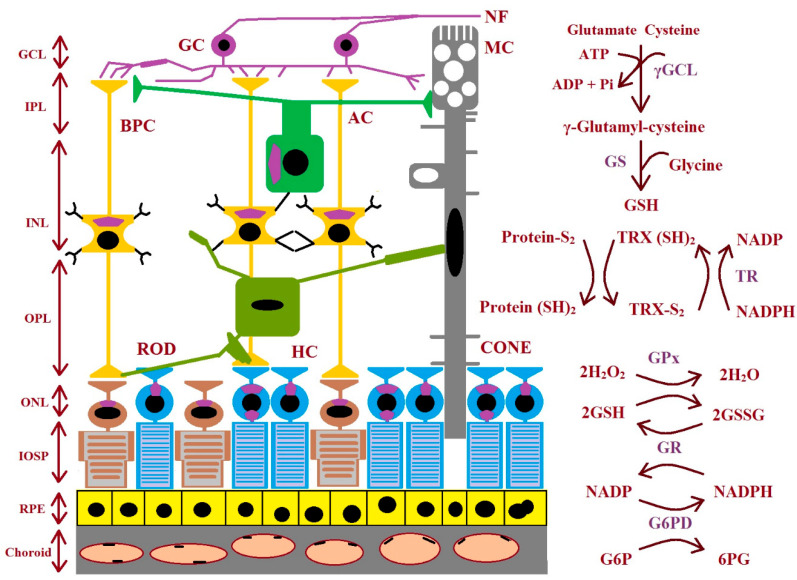
The figure shows the enzymes involved during glutathione synthesis and metabolism in the retina. AC: amacrine cell; BPC: bipolar cell; GC: ganglion cell; GCL: ganglion cell layer; γGCL: γ-glutamate cysteine ligase; G6PD: glucose-6-phosphate dehydrogenase; 6PG: 6-phosphogluconate; GPx: glutathione peroxidase; GR: glutathione reductase; GS: glutathione synthase; GSH: glutathione; ROD: rhodopsin; HC: horizontal cell; INL: inner nuclear layer; IPL: inner plexiform layer; IOSP: inner and outer segments of photoreceptors; NF: nerve fiber; ONL: outer nuclear layer; OPL: outer plexiform layer; protein-S2: protein-S−S; protein (SH)2: protein-SH; RPE: retinal pigment epithelium; TR: thioredoxin reductase; TRX (SH)2: thioredoxin; TRX-S2: oxidized thioredoxin (modified from the work of McBean et al. (2015) [48]).

GSH biosynthesis proceeds across many stages. There are three precursor amino acids (cysteine, glutamate, and glycine) that are combined to form GSH. This molecule is synthesized exclusively in the cytosol. This process requires the sequential action of two ATP-dependent enzymes: glutamate–cysteine ligase (GCL is composed of two subunits: a catalytic GCLC and a modifier GCLM) and glutathione synthetase (GS). GCL catalyzes the first stage of the biosynthetic pathway where glutamate and cysteine are linked, creating γ-glutamyl-cysteine in the presence of ATP and Mg^2+^ or Mn^2+^. The last stage of GSH synthesis involves the addition of glycine to γ-glutamyl-cysteine, which is catalyzed by GSH synthetase (Figure 3). The hydrolysis of GSH can be conducted by γ-glutamyl cyclo-transferase and cation transport regulator-like protein-1 (CHAC1) to obtain cysteinyl glycine and 5-oxoproline. Then, the breakdown of 5-oxoproline (which is catalyzed by 5-oxoprolinase) creates glutamate, and cysteinyl glycine is split by its respective peptidases to yield cysteine and glycine. These newly liberated amino acids can be reused for the synthesis of GSH [47,48]. The enzymes involved in GSH synthesis and metabolism in different types of retinal cells are shown in Figure 3.

As we said earlier, ROS are important in the development of AMD [2,3,4,8]. Therefore, research focusing on glutathione shows that this molecule plays a major role in protecting RPE cells against oxidative stress [36,48,49,50]. In the retina, GSH protects tissue against ROS because it is a thiol reductant and is able to detoxicate photooxidation products [42]. Studies have confirmed the role of GSH supplementation in preventing AMD [50,51,52]. On the other hand, GSH deficiency leads to the death of RPE cells. Unfortunately, the mechanism of increasing oxidative stress in RPE cells as a result of GSH depletion is still unknown [50]. 

Research has shown different results in terms of the concentration of GSH in the serum of patients with AMD compared to the controls. Some research has not indicated differences in the GSH levels between patients and healthy people [53,54]. Most studies showed decreased GSH levels in AMD patients compared to people without the disease [47,55,56,57,58,59,60]. There was even a study in which higher serum GSH concentrations were observed in AMD patients than in the control group [61]. These different results show that GSH is a non-specific parameter when analyzing the development of AMD. 

Cellular GSH status in RPE cells is decreased, which has been confirmed in many studies [50,51,62,63,64,65,66]. Only a few studies showed increased cellular GSH levels [65,67]. These novel studies concentrate on mitochondrial GSH (mGSH) because this molecule reaches a 5-10-fold greater concentration in this organelle than in the cytosol [47,68]. GSH is transported to the mitochondria via an efflux pump and takes part in antioxidant defense, the detoxification of xenobiotics, the stabilization of mDNA [47,69], the synthesis of cluster Fe-S (mGSH is a cofactor in this reaction) [47,70], and the electron transport chain (mGSH is a redox regulator) [47,71,72]. Studies on RPE cells showed that mGSH concentration is lower in the development of AMD [47,65,73,74].

## 5. Enzymes Engaged in the Maintenance of Functions of Glutathione

In cells, reduced glutathione (GSH) reacts with oxidants spontaneously or in reactions catalyzed by glutathione peroxidase (GPx) [35,39]. In effect, it creates glutathione disulfide (GSSG), which is composed of two glutathione molecules linked by a disulfide bridge. GSSG can be actively transported out of the cell or react with a protein sulfhydryl group (PSH). As a result of this process, a mixed disulfide (PSSG) is formed, but cellular GSH undergoes depletion [75]. GSSG can be reduced to GSH via glutathione reductase (GR) with the participation of 1 NADPH [24,42]. To protect proteins from harmful oxidative reactions, GSH insult trans-sulphuration reactions catalyzed by gluta-redoxin (GRX) [56,76]. On the other hand, glutathione transferase (GST) can conjugate GSH with many hydrophobic and electrophilic molecules (e.g., carcinogens, therapeutic drugs, and the products of oxidative metabolism), making them less toxic and easier to remove from cells [42,75]. 

Every fraction of the tetramer GPx contains a selenium atom [35]. This enzyme has eight isoforms distributed across different tissues (GPX1-GPX8) [77,78,79]. These isoforms are shown in Table 2. In this family, GPx1 is the most abundant member. This isoform is present in every cell and also in the cytosol, mitochondria, and peroxysomes. In some cells, it has been found in the peroxisomal compartment. This isoform plays a crucial role in preventing the harmful reaction of intracellular hydrogen peroxide and is much more effective than catalase [80]. Isoforms GPx1 and GPx4 probably have a protective role in the development of AMD [81,82]. In studies focusing on Polish patients with AMD, mutations in GPx1 were found, and these mutations lead to weaker antioxidant defense. This may have led to the development of AMD in this group of patients [81]. However, studies on mice showed that GPx4 suppresses increasing levels of vascular endothelial growth factor A (VEGF-A), which increase during neovascularization [82]. Studies also showed that lower GPx activity may increase the risk of developing AMD [83]. In several pieces of research, AMD patients were characterized by lower GPx activity [4,84]. Unfortunately, in recent AMD studies, patients had higher GPx activity than that seen in a healthy group [85].

Most studies on AMD showed that during this disease, GR activity was decreased [83,84,86] or that lower activity may increase the risk of developing AMD [83]. Research suggests that lower GR activity is associated with weaker antioxidant abilities [84]. There are some mechanisms relating to ganglion cell death during macular degeneration, including high intraocular pressure (IOP) and hypoxia, oxidative or nitrative stress, glutamate toxicity, loss of neurotrophic factors, and autoimmune reactions assisting in the degeneration of retinal ganglion cells. The retina is a highly vascularized tissue in the body and is vulnerable to oxidative stress due to its high consumption of oxygen and exposure to light. This tissue has one of the highest oxidative metabolic rates per tissue mass in the human body. In effect, it is important for retinal cells to maintain normal GSH levels and oxidation states. This is due to the fact that a deficiency of this molecule manifests in increased susceptibility to oxidative stress, and the resulting damage is thought to be a key step in the onset and progression of eye diseases [48]. 

The superfamily of GST enzymes is divided into cytosolic and microsomal groups, and the cytosolic family contains four groups: GSTA (α), GSTM (µ), GSTT (θ), and GSTP (π) [87]. The GSTM isoform is responsible for detoxifying ingredient electrolytes, such as drugs, carcinogens, environmental toxins, or products of oxidative stress [88,89]. GSTT catalyzes the combination of GSH with hydrophobic and electrolyte compounds. The main function of this isoform is to neutralize reactive low-molecular hydrocarbons, such as ethylene oxide [90]. Studies have shown that this isoform of GSTP protects the eye from oxidative stress. This protein can bind zeaxanthin and meso-zeaxanthine in the retina, suggesting that it can modulate antioxidant levels in the human eye [91,92]. 

## 6. The Role of Polymorphisms in AMD

It has been confirmed that no single gene is responsible for the development of AMD. As we stated in the beginning, AMD is a multi-factor disease. There are many genetic factors engaged in the development of AMD. However, the progression of this disease is determined in 50–60% of cases by the polymorphisms in three genes that encode the following: CFH (*rs1061170*), ARMS2 (*rs10490924*), and Il-8 (*rs2227306*) [93]. Other polymorphisms are less likely to correlate with the development of AMD, but their influence has been confirmed in other studies [81,93,94,95,96,97,98,99,100,101,102,103,104]. Genes and their polymorphisms are presented in Table 3. 

**Figure 4:**
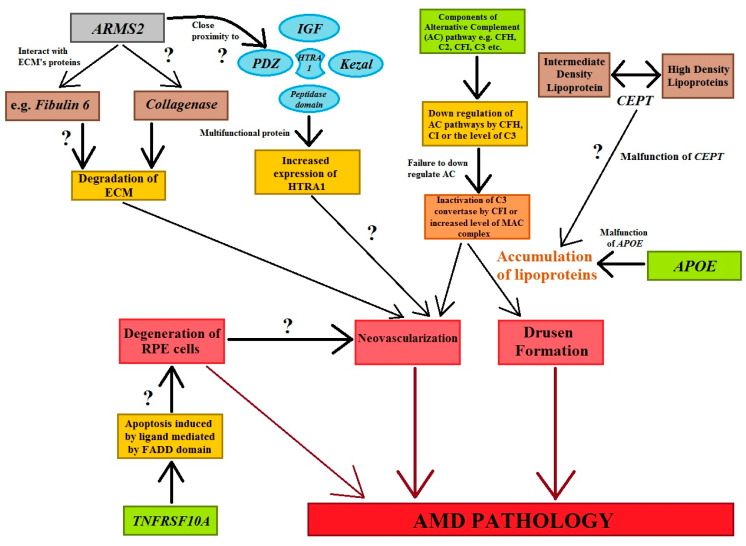
The figure shows the genes and their associations with AMD pathogenesis. Cellular functions, e.g., apoptotic, tumorigenesis, homologous recombination, angiogenesis, and inflammation are regulated by genes that stimulate the cardinal features of AMD abnormalities. Question marks symbolize the expected role of factors in the processes involved in AMD’s development (modified from the work of Anand et al. (2016) [95]).

The activation or dysregulation of the complement factors of the alternative complements pathway, like complement factor H (CFH), complement component (C2), complement factor I (CFI), and complement factor B (CFB), have been found to be associated with the development of AMD. Their influence is caused by the release of local inflammatory-activating products [93,95]. Complement factors, especially alternative complement pathway genes, have been reported in the pathogenesis of AMD (Figure 4). The data analyzed concerning four SNPs, *rs9332739* and *rs547154* for the *C2* gene and *rs4151667* and *rs641153* for the *CFB* gene, probably suggested that these alleles lower the risk of all AMD pathogenesis in the Caucasian population by 2.0% to 6.0%. It has been observed that the CFB polymorphism (*R32Q*) is greatly correlated with early AMD; on the other hand, it has a protective effect against late AMD in the Caucasian population [95,105]. It has been reported that *CFH* and *ARMS2* SNPs are significantly correlated with early AMD pathogenesis, and it has also been suggested that the polymorphisms of Apolipoprotein E (APOE) are associated with early AMD [106]. Tumor necrosis factor receptor superfamily 10A (TNFRSF10A) signaling plays a crucial role in apoptosis in cells. It has been identified as an important risk factor that is significantly correlated with AMD pathology. Studies focusing on Japanese populations showed a significant association between disease susceptibility and the *TNFRSF10A* gene on chromosome 8p21 (*rs13278062*) [95].

Human hepatic lipase (HL) plays an important role in lipid metabolism. This enzyme converts intermediate-density lipoproteins to low-density lipoproteins and is encoded by the *LIPC* gene. Polymorphism in this gene has shown strong evidence for its relationship with the pathogenesis of AMD [95,96]. Anand et al. (2016) [95] conducted studies and found the protective effect of the *TT* genotype in *LIPC*. Their work further revealed that SNP variation exists in the promoter region (*rs1046817*). This change influences *LIPC* expression, showing a strong association with AMD pathogenesis. The same study showed *LIPC* variance *rs493258* and its connection with the development of AMD [95]. A meta-analysis conducted in 2015 showed the protective role of the *rs10468017* polymorphism in the *LIPC* gene. This mutation elevates the level of HDL, which protects against oxidative stress. Another polymorphism in this gene engaged in a lipid metabolism pathway associated with the development of AMD was confirmed in gene *CETP* [96,97]. This gene encodes cholesteryl ester transfer protein (CETP), which regulates the concentration of cholesteryl esters in HDL molecules [97]. It was confirmed in a meta-analysis that *rs3764261* in *CETP* was associated with an increased risk of developing AMD. In theory, CETP shuttles triglyceride particles from very-low-density lipoproteins (VLDL) and LDL to HDL and creates triglyceride-enriched HDL. In effect, excessive HDL can lead to the accumulation of oxidized lipids in the retina, which is related to the development of AMD [96]. 

The age-related maculopathy susceptibility 2 (*ARMS2*) gene has been studied widely and replicated in several ethnic groups worldwide (Figure 4). *ARMS2* is predominantly located in high-energy-demanding tissues, like human photoreceptor cells. SNP variation in *ARMS2* genes (*LOC387715*) could reduce the stability of ARMS2 mRNA (Figure 4) [95]. HTRA1 are serine proteases composed of four protein domains for the binding of the insulin-like growth factor binding domain (ILGF), kazal domain, trypsin-like peptidase domain, and PDZ domain to accomplish cellular functions. Moreover, polymorphisms in the *ARMS2/HTRA1* (*rs10490924* and *rs11200638*) locus are significantly associated with early and late AMD; however, instead of this locus, complement factor components (C2, CFH) and complement factor B (CFB) have not shown a positive correlation with AMD abnormalities [95] (Figure 4). 

Multi-domain protein DICER1 is an important enzyme in the synthesis of short interfering RNAs (siRNAs∼21–25 nucleotides) from pre-double-stranded RNAs during RNA interference. DICER1 is the miRNA-processing enzyme required for the maturation of miRNAs. If there is any defect in DICER1, then most of these RNAs cannot be generated [107]. About 30% of human genes are regulated by micro-RNA classes of small RNA [95]. However, DICER1 plays an important function in visual activities by degrading toxic RNA. The accumulation of transcripts of Alu RNA in geographic AMD was caused due to dysregulation in DICER1. It has been reported that the accumulation of transcripts of Alu RNA in the geographic AMD was caused by the dysregulation of DICER1. DICER1 was found to be reduced in the advanced form of AMD, i.e., geographic atrophy in AMD patients [105].

The first study on the GST polymorphism in AMD patients was conducted in 2006; the research team analyzed DNA from the blood of patients to determine changes in this gene. The results suggested that the combinations of *GSTM1* (null) and *GSTP1* (mutant), *GSTM1* (null) and *GSTT1* (null) can be risk factors for the development of wet AMD [99]. Further studies on a larger number of individuals have shown that polymorphisms in this gene may correlate with the development of AMD. Studies confirmed that the *GSTM1* (null) polymorphism correlates with both dry and wet AMD stages [100]. In 2012, a Chinese research team analyzed the role of polymorphisms in the *GSTP1* gene (*rs1695* and *rs1138272*) in the development of wet AMD stage. The results showed that only *rs1695* moderately increases the risk of developing this disease [104]. In the same year, a GST polymorphism study was conducted in Iran [100]. The results showed that *GSTM1* and *GSTT1* correlate with a higher risk of the progression of AMD. The *GSTM1* polymorphism is associated with a decline in the expression of the protein, which is a risk factor. If the concentration or activity of this enzyme is lower, ROS cannot be effectively neutralized, affecting the development of AMD [98]. Unfortunately, cohort studies conducted in 2012 did not confirm the role of polymorphisms in genes *GSTM1*, *GSTT1*, and *GSTP1* in the development of AMD [108]. Studies conducted in 2016 showed that the control group was characterized by higher copy numbers of *GSTM1* than AMD patients. Moreover, it was the first report to show a lack of GSTM1 (null) (0/0) or polymorphisms of *GSTM5* (+/0, 0/0) in AMD. The authors suggested that weaker antioxidant functions could intensify oxidative stress and lead to the development of AMD [101]. 

Unfortunately, a meta-analysis conducted in 2021 showed no significant associations between *GSTM1/GSTT1* polymorphisms and the development of AMD in a Caucasian population [102]. Additionally, a meta-analysis conducted in 2022 showed no significant role of polymorphisms of *GSTM1* (*rs1183423000*), *GSTT1* (*rs1601993659*), and *GSTP1* (*rs1695*) in the development of AMD [103]. Only one study on the polymorphisms in gene *GPx* confirmed the influence of mutations in this gene on the development of AMD. A study on a group of Polish patients showed the polymorphism of *Pro197Leu* in the GPx1 isoform. This mutation is probably related to a weaker ability to scavenge ROS (especially hydrogen peroxide) in the retina, which may lead to the development of AMD [81].

## 7. Summary and Conclusions

Glutathione is the most important antioxidant in the eye. To maintain GSH function, an organism needs enzymes such as GPx, GST, GR, and GS. The proper functioning of enzymes and maintaining the balance between oxidants and antioxidants help avoid the negative effects of oxidative stress in the eye, which may lead to the development of many eye diseases. Numerous studies have found the exact role of glutathione in the development of AMD. Studies conducted on the activity of enzymes have shown that patients are characterized by lower GR activity. GST studies clearly showed an increase in activity in sick people, but for GPx activity, the results are not so clear. The results also suggest higher and lower GPx activity in patients with AMD. The improper functioning of enzymes may result from polymorphisms in their genes. Numerous studies have examined polymorphisms that can be associated with the development of AMD. The analysis of the polymorphisms in the GST genes confirmed that mutations lead to a weaker antioxidant barrier and may be conducive to the development of AMD. Non-specific results concerning the many parameters that make up the glutathione system reveal many unknowns. Thus, it is important to conduct further research to understand the exact mechanisms of the defensive functions of glutathione against oxidative stress in the human eye.

## Figures and Tables

**Table 1:** The table shows the main reactive oxygen species (ROS) and products of lipid peroxidation connected with oxidative stress in the eye (* symbolize free electron in molecule) [15,16,17,18,19].

ROS/Chemical Compounds	Characteristics and Activity
Superoxide radical anions (O_2_*^−^)	Is a product of the single-electron reduction of oxygen. O_2_*^−^ reacts relatively fast with compounds that contain thiol groups (like cysteine) or with such reductive compounds as ascorbate or NADH. It is also able to react quickly with metal ions (especially copper) or certain metalloproteins. O_2_*^−^ reactions are biologically important and lead to the reduction of transition metal ions (Fe^3+^ to Fe^2+^; Cu^2+^ to Cu^+^).
Hydrogen peroxide (H_2_O_2_)	Generated as a consequence of reactions of O_2_*^−^ molecules that are mediated by superoxide dismutase or non-enzymatically. It is a relatively stable compound but may undergo a disproportionation reaction catalyzed by transition metal ions. H_2_O_2_ is not excessively reactive. This molecule with superoxide radical anion (O_2_*^−^) takes part in Fenton and Haber–Weiss reactions and creates a hydroxyl radical (*OH).
Hydroxyl radicals (*OH)	These are some of the most reactive oxidative agents. They are able to react with practically all substances that are present in an organism; however, in lots of cases, the tempo of such reactions is determined by diffusion only (not by an activation barrier). The biologically critical consequence of *OH reactivity is a risk factor for the generation of DNA damages. *OH is produced in Fenton and Haber–Weiss reactions or as a by-product from the process of water radiolysis.
Products of LPO	Characteristics and Activity
Malondialdehyde (MDA)	It is a reactive aldehyde generated as a result of polyunsaturated lipid degradation by ROS. MDA constitutes a highly toxic and mutagenic compound that forms lipoperoxidation end-products. It is capable of reacting with lysine residues on proteins, leading to the formation of secondary oxidation products. It may also participate in provoking DNA damages (reaction with deoxy-guanosine of DNA and the generation of 8-hydroxy-2-deoxy-guanosine) or interact with deoxyadenosine residues.
4-Hydroxynonenal (4-HNE)	This aldehyde compound is highly reactive and able to form adducts with cellular proteins and DNA. 4-HNE is mainly produced by such precursor substances as arachidonic acid and linolenic acid through 15-lipoxygenase. Electrophilic double-bond and nucleophilic amino acid residues on proteins enable 4-HNE to form Michael adducts with such amino acids as cysteine, histidine, and lysine residues. It leads to increases in their molecular masses (by the molecular mass of HNE 156 Da). These adducts are relatively stable and capable of remaining in cells even for several hours, provoking dysfunctions of the targeted biomolecules.

**Table 2:** The table shows the isoforms of glutathione peroxidase (GPx), their location, and role [79].

Isoform	Location	Role
GPx1	Every cell (cytosol, mitochondria, and peroxysomes)	Plays a major role in protecting against systemic oxidative stress.
GPx2	Gastrointestinal system	In mammals, it is the first barrier that prevents the absorption of hydroperoxides produced by food.
GPx3	Plasma	In the plasma, it is a major ROS scavenger, eliminating all circulating complex hydroperoxides.
GPx4	Cell membrane	It is able to reduce and destroy lipid hydroperoxides.
GPx5	Epididymal tissues	In the epididymis, it regulates oxidative stress damage and protects sperm maturation.
GPx6	Embryonic and olfactory organ epithelial cells	It probably plays a role in the transmission and degradation of odor-related signals.
GPx7	Endoplasmic reticulum	It plays a unique role in maintaining redox homeostasis. It has different responses to oxidative stress than other enzymes from the GPX family.
GPx8	Transmembrane protein	It exerts a role in oxidative stress, takes part in folding endoplasmic reticulum proteins and regulates the concentration of Ca^2+^ in the endoplasmic reticulum.

**Table 3:** The table presents an overview of the polymorphisms engaged in the development of AMD.

Gene	Polymorphism	Mechanism	Reference
*CFH*	*rs1061170 (Y402H)*	Hyperactivation of the alternative complement pathway	[93]
*ARMS2*	*rs10490924*	Changes in the function of retinal mitochondria	[93]
	*LOC387715*	Reduction in ARMS2 mRNA stability	[93,94]
*Il-8*	*rs2227306*	Increase in inflammation and angiogenesis	[93]
*LIPC*	*rs1046817*, *rs493258*, *rs10468017*	Changes in HDL levels	[95]
*C2*	*rs9332739, rs547154*	Lowering the risk of progression to late AMD	[93,95]
*CFB*	*rs4151667, rs641153*	Lowering the risk of progression to late AMD	[93,95]
*TNFRSF10A*	*rs13278062*	Enables apoptosis in cells	[95]
*HTRA1*	*rs11200638*	Elevates the expression of HtrA1	[95]
*CETP*	*rs3764261*	Changes in the concentration of cholesteryl esters in HDL	[96,97]
*GSTM1*	*rs1183423000*	Weaker antioxidant protection	[98,99,100,101,102,103]
*GSTT1*	*rs1601993659*	Weaker antioxidant protection	[99,102,103]
*GSTP1*	*rs1695*, *rs1138272*	Weaker antioxidant protection	[99,103,104]
*GPx1*	*Pro197Leu*	Weaker ability to scavenge hydrogen peroxide	[81]
